# Arterial blood analysis of healthy residents in Huamachuco, Peru (3,164 m): a cross-sectional study

**DOI:** 10.12688/f1000research.134567.2

**Published:** 2023-09-26

**Authors:** Victor H. Bardales-Zuta, Lissett J. Fernández-Rodríguez, Cecilia Romero-Goicochea

**Affiliations:** 1 Physiology Laboratory, Medical School, Universidad Privada Antenor Orrego, Trujillo, La Libertad, Peru; 2 Hospital Florencia de Mora-ESSALUD, Trujillo, Peru

**Keywords:** blood gas, altitude, Andean, blood pH, blood calcium, blood glucose, hematocrit, Huamachuco

## Abstract

**Background**: Given that arterial blood gas is affected by altitude and ethnicity, establishing reliable reference standards for these values requires analysis of arterial blood at different elevations and locations. Our objective was to measure the arterial blood gases of healthy young volunteers in Huamachuco, Peru, at 3,164 m above sea level. This is likely the first study of arterial blood gas analysis of healthy Northern Peruvians living at high altitude.

**Methods**: Healthy residents of Huamachuco were recruited for this cross-sectional convenience sample study and arterial blood was drawn by standard procedures. People with obesity, diabetes, high levels of physical activity and a history of using selected substances were excluded. The samples were analyzed on-site in less than 15 minutes using a Stat Profile Prime CCS analyzer (Nova Biomedical).

**Results**: Data from 46 participants (17 male, 29 female) were included in the study. The median values for arterial blood pH, oxygen, carbon dioxide, ionized calcium, glucose, lactate, hematocrit, oxygen saturation, and bicarbonate were 7.42, 9.3 kPa (70 mmHg), 4.5 kPa (33.5 mmHg), 1.04 mM, 5.19 mM, 1.8 mM, 50 %, 94 %, and 21.6 mM, respectively. We also found a lower prevalence of diabetes among highlanders compared to the Peruvian population.

**Conclusions**: The results determined here were comparable to other results determined at different altitudes in the Americas, although arterial blood oxygen was slightly higher than predicted. These results indicate that Northern Peruvians have an Andean-style adaptation to high altitude.

## Introduction

Approximately 25.2 million people live at an altitude greater than 3,000 meters above sea level, in an environment marked by decreased atmospheric pressure ^1^ which causes a series of downstream physiological effects that must be compensated to maintain homeostasis. Arterial blood gas analysis (ABGA) offers a window into these mechanisms for residents and sojourners at different altitudes.

ABGA is also an important tool for monitoring and diagnosing cardiopulmonary disfunction, which requires comparison of reference values to a result. ^2^ However, establishing reference intervals is a significant challenge, especially for ABGA at high altitude, for three reasons: (1) few studies have examined reference intervals among healthy people because the test is frequently ordered for unwell patients, ^2^^,^^3^ (2) obtaining arterial blood is a more difficult procedure that causes more pain to the patient than routine venous phlebotomy, ^4^ and (3) different ethnic groups have different compensating mechanisms for life at high altitude: for instance, South American natives adapt by increasing hemoglobin, while Tibetan natives improve blood circulation. ^5^^,^^6^

To provide additional ABGA values at high altitude and to better understand altitude adaptation mechanisms between ethnic groups, we performed arterial blood analysis on young healthy volunteers resident in Huamachuco, Peru (elevation 3,164 m) and compared them with published results at a variety of elevations and locations. We hypothesized that residents in Huamachuco will exhibit similar adaptations seen in other Andean high-altitude natives, regardless of biological sex. ^5^^,^^6^

## Methods

### Ethics statement

Although the research took place at Instituto de Educación Superior Tecnológico Público de Huamachuco (IESTPH) due to its high-altitude location, none of the authors are affiliated with IESTPH, and IESTPH does not have, to the best of our knowledge, an ethics committee capable of approving the study. Because of this, The Training, Teaching and Research Committee (ethical approval committee) of Florencia de Mora – ESSALUD Hospital I approved this study, on 01 April 2019. We sought approval from the Florencia de Mora Hospital as the first author is a doctor based at this hospital. The administration of IESTPH was informed of the project with a formal letter. The National University of Trujillo Postgraduate School also ethically approved this study; VHBZ was affiliated with that institution at the time of the study.

Volunteers were informed of the risks of arterial blood sampling and provided their written informed consent for arterial blood sampling and for the publication of the results. Participation in the study was strictly voluntary with no benefits provided, except for personal analysis results. There was one participant that was under the age of 18 (17 years old). In this case, the participant and the participant’s father signed the consent form for use with minors.

This cross-sectional convenience-sample study was not preregistered.

### Study location

IESTPH was chosen for the study site because it was high-altitude location that could accommodate the equipment necessary for arterial blood analysis. The GPS coordinates and altitude of the study location were measured using a Samsung Galaxy A3 with the “Precise Altimeter” application.

### Recruitment of participants

A letter from the director or IESTPH was sent to students of the same institution inviting them to participate in the study. Recruitment took place between May to August 2019. On the day of the study, August 26, 2019, the fasting (>8 h) volunteers were informed of the risks of arterial blood sampling. Written informed consent for arterial blood sampling and for the publication of the results was obtained from all participants. Although the study took place in 2019, we are publishing this work now due to delays caused by the COVID-19 pandemic.

### Inclusion and exclusion criteria

Inclusion criteria for the entire study were age between 17 and 30 years and residence in Huamachuco district for at least 5 consecutive years prior to the study and no recent travel to low-altitude locations. Exclusion criteria included self-report of strenuous exercise more than 60 minutes per day, use of tobacco, antiplatelet agents, anticoagulants, diuretics, corticosteroids, beta-blockers, or some beta-stimulants, or recent travel to low elevations. Exclusion criteria also included self-report or clinical signs upon evaluation of cardiovascular, pulmonary, or hematologic disease. Participants with self-reported diabetes and/or diabetes determined by arterial blood test (defined here as arterial blood glucose greater than 7.2 mM to account for the arteriovenous glucose difference ^7^^,^^8^), had BMI >30, or had abnormal axillary temperature (not between 35.5 and 37.0°C ^9^) were allowed to continue their participation, but their ABGA results were not used in the aggregated data. However, data from participants with diabetes was combined with other participant data to estimate diabetes prevalence.

### Procedure

Volunteers were questioned by a nurse from IESTPH to determine whether the participant met inclusion/exclusion criteria. The nurse measured the height, body mass, pulse rate, blood pressure, and axillary temperature of each participant. Volunteers were also asked to self-report their biological sex. Body mass index (BMI) was calculated by dividing body mass by the square of the height. If the volunteers wished to continue, physicians (authors VHBZ and/or LJFR) obtained one sample of arterial blood (1 mL) from the right brachial artery of the volunteers using standard sterile technique and a heparinized needle (Westmed Pulset 3cc syringe). Blood samples were stored on ice in a cooler prior to analysis.

Personnel from a commercial laboratory (BermanLab, Trujillo, Perú) analyzed blood gas parameters of the samples at IESTPH less than 15 minutes after sampling to avoid contamination during storage and transport. The blood gas analyzer (Stat Profile Prime CCS, Nova Biomedical) passed operational qualification in Huamachuco and was used to measure pH, partial pressure of oxygen (pO _2_) and carbon dioxide (pCO _2_), plasma ionized calcium (iCa ^2+^), glucose (Glc), lactate (Lac), and hematocrit (Htc, %). Bicarbonate (HCO _3_^−^) and oxygen saturation (sO _2_) were calculated from the measured parameters.

### Bias

The most likely experimental source of bias in this experiment is air contamination of the sample before or during analysis. Air contamination of the sample would artificially elevate pO _2_ without causing large changes in other parameters. This bias was addressed by careful sampling technique, rapid analysis of the sample, and removal of suspiciously high pO _2_ measurements by statistical methods. A second source of possible bias is due to the inclusion of unhealthy individuals in the ABGA, such as those with diabetes, obesity, or abnormal axillary temperature. If ABGA results were from a patient with diabetes, obesity, or abnormal axillary temperature or if the ABGA results were suspected of air contamination, the results were eliminated from the aggregated arterial blood analyses. However, these results were included in our analysis of the prevalence of diabetes and impaired fasting glucose.

### Statistical analysis

ABGA results with pO _2_ outlier values were eliminated from the dataset (Tukey’s fence, k = 1.3). Shapiro-Wilk normality tests, Mann Whitney U tests, and linear regression analyses were used to analyze the dataset, compare different groups, and compare these results with previously reported data at a significance level of *p* < .05 using a Bonferroni correction where appropriate. Quantitative variables were reported as means and standard deviations or by quartile depending on the distribution. If the Mann Whitney U test revealed a significant difference between the sexes, data was reported for males and females separately. Confidence intervals were calculated using the t-distribution for ABGA parameters, and the Wilson score interval was calculated for diabetes prevalence values. We used Microsoft Excel for Mac version 16.73 and R version 4.3.0 to analyze the data. Since all participants completed a single blood test on the same day, there was no missing data.

## Results

### Study location

Huamachuco district has an area of 424 km ^2^, and a 2017 population of 66902 including 16456 17 to 30 year old inhabitants. ^10^ A topographic analysis of the district indicates that it varies from 2,200 to 4,600 m, but most of the population lives below 3,400 m. The largest city, also named Huamachuco, is located at about 3,200 m. IESTPH, where the samples were taken, is located at 7.815833 S, 78.03917 W and had a GPS altitude of 3,164 m.

### Demographic data of the participants

The study size was based on available IESTPH students that volunteered and met acceptance criteria. A total of 56 participants (21 male and 35 female) volunteered for the study. Ten participants (4 male and 6 female) were excluded from ABGA after blood sampling because they had diabetes, unusual body temperature, obesity, or outlier arterial pO _2_. Some participants excluded at the interview, axillar temperature, or by excessive BMI freely decided to have their blood sampled and analyzed to know their results.

The 46 participants included in our ABGA analysis were between 17 and 28 years old, with a median age of 20 years. Ages were skewed toward lower values: the Shapiro-Wilk tests showed a significant departure from normality for age, *W*(46) = .885, *p* < .001. Body height was greater in males than females (Mann Whitney U test, *p* < 0.001): females had a median height of 1.51 m, and males had a median height of 1.60 m. Statistically significant differences between the sexes in other anthropomorphic parameters were not found. Body mass index had a normal distribution (Shapiro-Wilk, *W*(46) = .97, *p* = .31) and had an average of 23.4 and a standard deviation of 2.8. No participant had a BMI > 30, high blood pressure or diabetes. The median blood pressure was 100/60 mmHg, with a variation of less than 20 mmHg. The median pulse rate was 67 with a range of 55 to 89 min ^-1^. Most of the participants had an axillar temperature of 36.6°C, but the range was 36.0 to 37.0°C.

### Blood gas parameters of the participants

The summary values of the resulting analyses are recorded in Table 1.

**Table 1.** ABGA results for 46 healthy young adults in Huamachuco (3164 m). Values are expressed as minimum, first quartile, median, third quartile, maximum and 95% confidence interval (95% CI) as calculated using the t-distribution, as some results showed a statistically significant departure from normality. Males and females are separated where a statistically significant difference was found.

Parameter	Minimum	First quartile	Median	Third quartile	Maximum	95% CI
pO _2_, kPa (mmHg)	8.0 (60)	9.0 (67)	9.3 (70)	9.6 (72)	10.4 (78)	8.94 – 9.49 (67.0 – 71.2)
sO _2_, %	91	93	94	94	96	93.3 – 94.4
pCO _2_, kPa (mmHg)	3.6 (27)	4.1 (31)	4.5 (33.5)	4.7 (35)	5.5 (41)	4.32 – 4.53 (32.4 – 34.0)
pH	7.38	7.40	7.42	7.43	7.45	7.410 – 7.422
HCO _3_^-^, mM, male	20.1	21.5	22.4	23.1	24.8	21.6 – 22.9
HCO _3_^-^, mM, female	16.7	19.9	20.9	21.9	22.7	20.2 – 21.3
iCa ^2+^, mM	0.95	1.01	1.04	1.06	1.13	1.04 – 1.08
Glc, mM	3.94	4.84	5.19	5.61	7.10	5.11 – 5.55
Lac, mM	0.8	1.5	1.8	2.3	3.9	1.72 – 2.10
Htc, %, male	51	53	54	55	60	53.2 – 55.8
Htc, %, female	40	46	47	48	54	46.0 – 48.3

### Differences between sexes for blood parameters

There were significant differences for Htc % (Mann Whitney U test, *p* < 0.001) and HCO _3_^−^ (Mann Whitney U test, *p* = 0.0019). Other ABGA parameters failed to reach statistical significance between sexes with Bonferroni correction.

### Correlation between the measured blood parameters

We compared 21 combinations between pH, pO _2_, pCO _2_, iCa ^2+^, Glc, Lac, Htc, and HCO _3_^−^ using regression analysis and Bonferroni correction. Two comparisons achieved statistical significance: (1) glucose (mM) predicted lactate (mM) (R ^2^ = .24, F(1,44) = 13.96, *p* < .001. β = .42, *p* < .001, α = -0.34, *p* = .57) and (2) pCO _2_ (kPa) predicted HCO _3_^−^ (mM) (R ^2^ = .71, F(1,44) = 107.16, *p* < .001. β = .378, *p* < .001, α = 4.58, *p* = .007). pH and pCO _2_ and pH and glucose had inverse relationships that were close to reaching statistical significance.

## Discussion

Living in the reduced atmospheric pressure of a high-altitude environment causes a series of physiological adaptations to maintain adequate blood oxygenation. These adaptations have been shown to vary between ethnic groups ^5^^,^^6^ making it necessary to sample different healthy populations to establish reference intervals. To our knowledge, this is the first systematic ABGA of healthy northern Peruvians living at high altitudes. Furthermore, this study includes analysis of arterial iCa ^2+^, Glc, and Lac, which are also not frequently measured among healthy high-altitude residents.

### pO _2_

**Figure 1.**
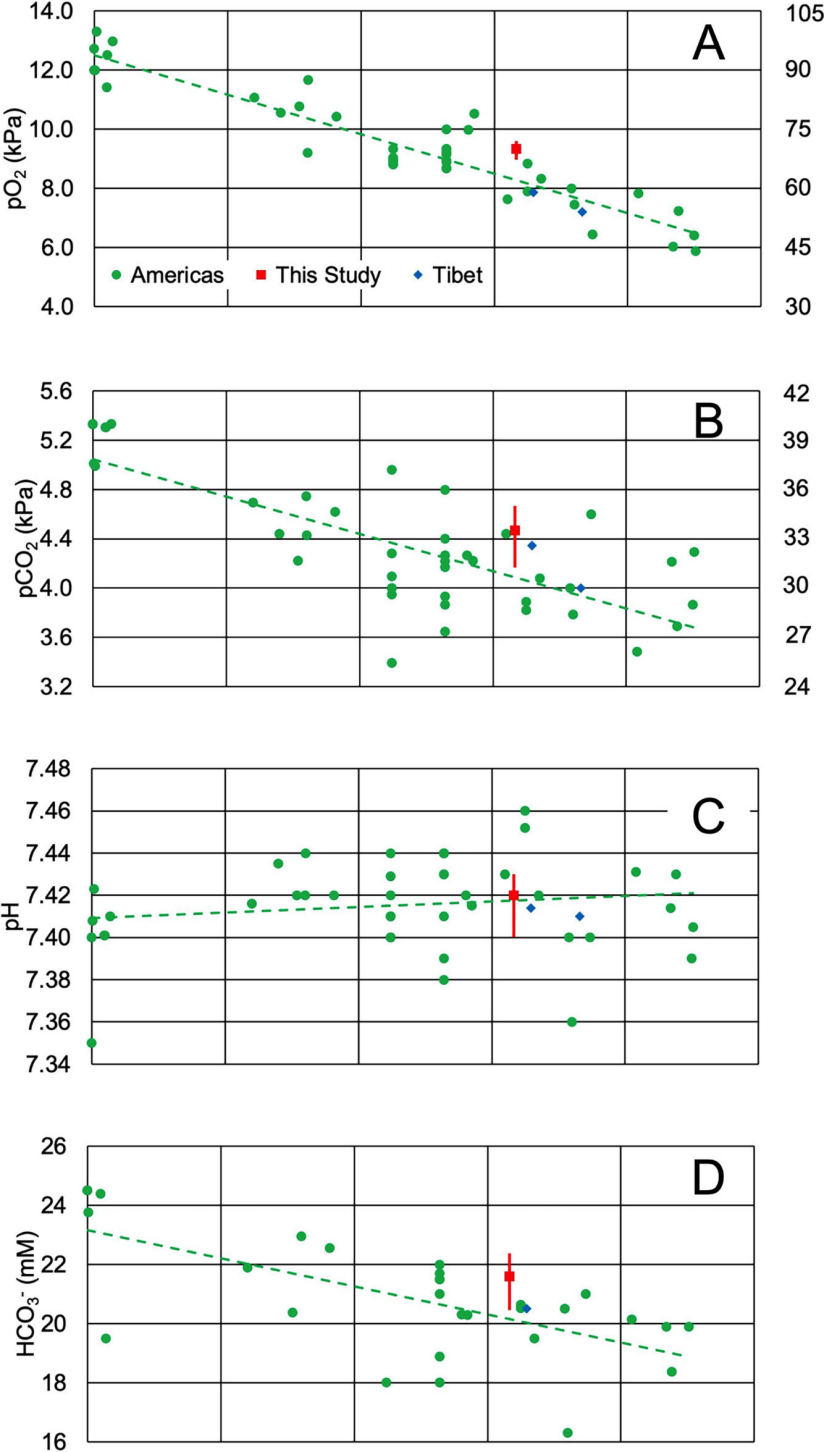
Correlations between altitude and various blood parameters. Each panel represents a different blood parameter and its relationship to altitude (meters above sea level): (A) pO _2_ (kPa), (B) pCO _2_ (kPa), (C) pH, and (D) HCO _3_^-^ (mM). Green circle data points are averages or medians from previously published results in the Americas; where possible, age range was considered to coincide with the age range in this study. ^11^^–^^30^ The red square data point is the median determined here, with whiskers representing the interquartile range. Data from native Tibetans are represented as blue diamonds. ^31^^,^^32^ Previously published results from the Americas (green circle data points) were used to calculate the regression lines in the figure and regression statistics in the text.

A growing body of ABGAs of healthy residents of the Americas at different elevations makes a systematic comparison of pO _2_ and altitude possible. Figure 1A visualizes the published pO _2_ for healthy residents of the Americas living at different altitudes. The results collected over the past half century ^11^^–^^30^ reveal that the inverse relationship between meters above sea level and pO _2_ (kPa) can be modeled with a linear equation up to about 4,500 m ( *R* ^2^ = 0.87, *F*(1,40) = 273.13, *p* < .001, β = -0.00133, *p* < .001, α = 12.5, *p* < .001). Inspection of Figure 1A reveals that there is a lack in published ABGA results between 100 to 1000 m. It is likely that data in this range will be useful in confirming or rejecting a linear model for pO _2_ and altitude. The median value of pO _2_ determined here is 1 kPa (7.50 mmHg) higher than the predicted value of the regression, although the data range overlaps the trendline and the point is within the spread of the data.

One possible explanation is that arterial blood oxygen decreases with age and the ages of the participants in this study were skewed toward young adults. ^2^^,^^11^ When possible, only subjects that resembled the ages of the participants were included in the regression, but selection criteria and age range reporting differed between studies; older participants in the published studies included in the regression would systematically lower the regression line ( Figure 1A). A second possibility was hyperventilation during sample acquisition, but breathing rate among participants was not monitored. Changes in equipment use over the years may also have added systematic errors to the data.

Andeans have been shown to have elevated oxygen saturation compared to Tibetan highlanders. ^5^^,^^6^ In fact, the results of this study confirm higher pO _2_ for Andean residents, as two studies that measured similar parameters in Tibet ^31^^,^^32^ fell slightly below the regression line ( Figure 1A, diamonds). Furthermore, the results from Tibet were approximately 2 kPa (15 mmHg) below our result. Therefore, these results suggest support for the hypothesis that Andeans have higher oxygen saturation than Tibetans at similar altitudes, although more experimental data from random population samples at different altitudes in Tibet and Peru more would help confirm this observation.

### pH and acid-base balance

Blood pH is tightly regulated through several buffering systems, the most important of which is the bicarbonate-carbonic acid system, which is derived from dissolved carbon dioxide. Like pO _2_, pCO _2_ (kPa) has an inverse relationship with altitude in m (R ^2^ = .57, F(1,40) = 53.27, *p* < .001. β = -.0003, *p* < .001, α = 5.05, *p* < .001, Figure 1B), although the slope of the correlation is less steep and altitude explains less of the variation in pCO _2_ than it does with pO _2_ (Compare Figure 1A and B). This decrease in pCO _2_ as altitude increases is usually explained by hyperventilation to compensate for decreased oxygen partial pressure at high altitude. We find that our results largely follow this trend. Unlike some other studies, ^2^^,^^12^^,^^33^ a statistically significant difference between sexes for pCO _2_ was not observed.

Although altitude and pCO _2_ were moderately correlated, no such relationship is present for pH, which remains almost constant regardless of altitude in m (R ^2^ = .025, F(1,40) = 1.01, *p* = .320, β = .00000264, *p* = .320, α = 7.41, *p* < .001, Figure 1C). The results determined here nearly exactly overlap the regression line. Given that dissolved CO _2_ has an inverse relationship with pH, the pH should also increase with altitude, but this is compensated by renal excretion of nonvolatile bases. ^34^^,^^35^ These changes also require modification of the Siggard-Anderson acid-base chart to account for altitude. Our data support moving the normal target of a plot of pH vs pCO _2_ downward, but the variance of our results suggests a widening of the vertical size of the target. ^34^

This increased bicarbonate excretion is also reflected in an inverse relationship between HCO _3_^−^ (mM) and altitude in m ( Figure 1D; R ^2^ = 0.43, F (1,27) = 20.13, *p* < .001, β = -.00095, *p* < .001, α = 23.16, *p* < .001). Since HCO _3_^−^ is derived from pH and pCO _2_, it follows that if pH is relatively stable, HCO _3_^−^ would also decrease if pCO _2_ also decreases. We found no correlation between blood pH and actual HCO _3_^−^ or pH and pCO _2_. This is likely because maintenance of pH within a narrow range result ultimately in a lower concentration of oxygen, carbon dioxide and bicarbonate, creating a new equilibrium at a lower buffer concentration but the same pH. A study at sea level did find weak correlations between HCO _3_^−^ , pH and pCO _2_, with higher pH having lower pCO _2_. ^2^ It is likely that this study was able to find these correlations because of its larger size, but the difference is over 0.1 pH unit. The HCO _3_^−^ concentration determined here is higher than the regression line, but the spread of the data overlaps the trendline, and males had statistically significantly higher HCO _3_^−^ than females.

### iCa ^2+^

Among healthy individuals, calcium is maintained within a narrow range. Departures from this range lead to several severe symptoms, including tetany, vomiting, coma, and neurological disturbances. ^36^ The reference interval for iCa ^2+^ is between 1.05 and 1.30 mM, which is slightly higher than the range (0.95 to 1.13 mM) found here. ^36^ Similar studies in the Andes also reported average results below the lower limit of the reference range. ^13^^,^^14^ Since iCa ^2+^ is dependent on several factors, including albumin content, vitamin D, and blood pH, it is not yet possible to determine whether lower iCa ^2+^ is a result of altitude or another confounding factor. Correcting iCa ^2+^ for pH did not change values significantly: the largest adjustment was not greater than 0.02 mM.

### Lac and Glc

Lac concentration is a measure of anaerobic respiration and tissue hypoxia and has a reference range of between 0.3-0.8 mM, but this has been called into question. ^36^^,^^37^ The range of values determined here is both wider and has a higher median than the reference range. A study at higher altitude also reported higher Lac. ^14^ These results may reflect a variety of activity levels among study participants, especially considering that the test site was in mountainous terrain. Alternatively, reduced oxygen at high altitude can promote anaerobic metabolism, but high-altitude residents have been shown to have Lac levels similar to those of lowlanders after exercise. ^38^ We found a positive correlation with Lac and Glc concentration, which may be because of their relationship in the Cori cycle, but this hypothesis requires further testing.

### Diabetes and impaired fasting glucose prevalence

Due to the selection criteria, only subjects with normoglycemia and reasonable ABGA results were included in the data analysis. However, it is unlikely that fasting arterial blood Glc would be impacted by outlier ABGA results, which provides an opportunity to measure diabetes and impaired fasting blood glucose (IFBG) prevalence in the entire study population of 56 people. Diabetes and IFBG are usually defined in terms of glucose concentration in venous blood plasma; arterial blood was measured here. For fasting individuals, the arteriovenous glucose difference is on the order of 0.2 mM, which revises the cutoff to ≥ 7.2 mM for diabetes and between 5.8 and 7.2 mM for IFBG. ^7^^,^^8^ Given these criteria, the prevalence was 2/56 (3.6%, 95% CI [0.98%, 12%]) for diabetes, and 10/56 (17.9%, 95% CI [10%, 30%]) for IFBG. Aggregated diabetes prevalence in Peru is approximately 6% and IFBG prevalence is approximately 15-20%. However, highland prevalence is lower for both diabetes and IFBG, where it is estimated to be approximately 4.5% and 17.4%, respectively. ^39^ These results are almost exactly what we observe as well, further supporting that living at high altitude correlates with lower diabetes prevalence.

*Htc.* Htc, a measure of the red blood cell content of blood, has been shown to vary between biological males and females, as well as between different high-altitude resident populations. Andeans and acclimatized Europeans tend to respond to high altitude by increasing hemoglobin and Htc% in a dose-response manner. ^5^^,^^6^^,^^40^ The hematocrit determined here ( Table 1) falls on the high end of the sea-level reference interval (42-52% and 37-47% for males and females respectively), suggesting similar adaptations seen in other Andean high-altitude natives. ^5^^,^^6^^,^^36^

## Conclusions

We observe that in resident Andean highlanders pO _2_ and pCO _2_ decrease, Htc% increases, and pH and iCa ^2+^ do not change very much with altitude. Thus, Northern Peruvians seem to have similar adaptations seen in other Andean high-altitude natives. However, these results show higher than expected pO _2_ and pCO _2_ for the altitude of the analysis. This is likely due to the young age range of the participants and the small study sample.

This study used a convenience sample cross section of young and healthy Andeans, which means it is not likely applicable to older people or those with chronic conditions, those with different altitude adaption profiles, or sojourners to the high-altitude environment. Additional sample taking of a more demographically balanced cross section and controls preventing air exposure of the sample are necessary to confirm these results and make the generalizable to the population of Huamachuco. Furthermore, comparing the results as a function of barometric pressure rather than altitude can eliminate some variability.

We hope that these results can be helpful in further establishing altitude- and ethnicity-dependent data-supported values for ABGA as well as Glc, Lac, and iCa ^2+^, which will aid in the diagnosis and treatment of cardiopulmonary disease at high altitude.

## Data Availability

Figshare: Dataset for “Arterial blood gas analysis of healthy residents in Huamachuco, Peru (3,164 m)”. https://doi.org/10.6084/m9.figshare.22720630. ^41^ This project contains the following underlying data:
• Huamachuco-calculations-110623.xlsx (Dataset for the article “Arterial blood gas analysis of healthy residents in Huamachuco, Peru (3,164 m)”. All raw data collected, most calculations, and results reported in the figure and table are included in this spreadsheet.) Figshare: STROBE Checklist for “Arterial blood gas analysis of healthy residents in Huamachuco, Peru (3,164 m)”. https://doi.org/10.6084/m9.figshare.22720723. ^42^ Data are available under the terms of the Creative Commons Zero “No rights reserved” data waiver (CC0 1.0 Public domain dedication).

## References

[1] Tremblay JC Ainslie PN : Global and country-level estimates of human population at high altitude. *Proc Natl Acad Sci U S A.* 2021;118(18):e2102463118. 10.1073/pnas.2102463118 33903258 PMC8106311

[2] Klæstrup E Trydal T Pederson JF : Reference intervals and age and gender dependency for arterial blood gases and electrolytes in adults. *Clin Chem Lab Med.* 2011;49(9):1495–1500. 10.1515/CCLM.2011.603 21619466

[3] Chandran J D’Silva C Sriram S : Clinical Utility of Arterial Blood Gas Test in an Intensive Care Unit: An Observational Study. *Indian J Crit Care Med.* 2021;25(2):172–175. 10.5005/jp-journals-10071-23719 33707895 PMC7922451

[4] Matheson L Stephenson M Huber B : Reducing pain associated with arterial punctures for blood gas analysis. *Pain Manag Nurs.* 2014;15(3):619–624. 10.1016/j.pmn.2013.06.001 24572291

[5] Beall CM : Andean, Tibetan, and Ethiopian patterns of adaptation to high-altitude hypoxia. *Integr Comp Biol.* 2006;46(1):18–24. 10.1093/icb/icj004 21672719

[6] Julian CG Moore LG : Human Genetic Adaptation to High Altitude: Evidence from the Andes. *Genes (Basel).* 2019;10:150. 10.3390/genes10020150 30781443 PMC6410003

[7] ElSayed NA Aleppo G Aroda VR : 2. Classification and diagnosis of diabetes: standards of care in diabetes – 2023. *Diabetes Care.* 2023;46(Suppl. 1):S19–S40. 10.2337/dc23-S002 36507649 PMC9810477

[8] Somogyi M : STUDIES OF ARTERIOVENOUS DIFFERENCES IN BLOOD SUGAR: I. EFFECT OF ALIMENTARY HYPERGLYCEMIA ON THE RATE OF EXTRAHEPATIC GLUCOSE ASSIMILATION. *J Biol Chem.* 1948;174(1):189–200. 10.1016/S0021-9258(18)57386-5 18914074

[9] Sund-Levander M Forsberg C Wahren LK : Normal oral, rectal, tympanic and axillary body temperature in adult men and women: a systematic literature review. *Scand J Caring Sci.* 2002;16:122–128. 10.1046/j.1471-6712.2002.00069.x 12000664

[10] Instituto Nacional de Estadística e Informática: *Departamento de La Libertad: Resultados Definitivos 2017.* Lima, Peru:2018. (accessed 19 September 2022). Reference Source

[11] Crapo RO Jensen RL Hegewald M : Arterial blood gas reference values for sea level and an altitude of 1,400 meters. *Am J Respir Crit Care Med.* 1999;160:1525–1531. 10.1164/ajrccm.160.5.9806006 10556115

[12] Gonzalez-García M Maldonado D Barrero M : Arterial blood gases and ventilation at rest by age and sex in an adult Andean population resident at high altitude. *Eur J Appl Physiol.* 2020;120(12):2729–2736. 10.1007/s00421-020-04498-z 32939642

[13] Tinoco Solórzano A Román Santamaría A Charri VJ : Gasometría arterial en diferentes niveles de altitud en residentes adultos sanos en el Perú. *Horiz Med.* 2017;17(3):6|–10. 10.24265/horizmed.2017.v17n3.02

[14] Viruez-Soto JA Jiménez-Torres F Sirpa-Choquehuanca V : Gasometría arterial en residentes a gran altura, El Alto – Bolivia 2020. *Cuad Hosp Clín.* 2020;61:38–43.

[15] Weil JV Jamieson G Brown DW : The red cell mass-arterial oxygen relationship in normal man: application to patients with chronic obstructive airway disease. *J Clin Inv.* 1968;47:1627–1639. 10.1172/JCI105854 5658592 PMC297320

[16] Torrance JD Lenfant C Cruz J : Oxygen transport mechanisms in residents at high altitude. *Resp Physiol.* 1970;11:1–15. 10.1016/0034-5687(70)90098-8 5491113

[17] Restrepo J Reyes P Vasquez P : Gasimetria arterial y alveolar en adultos sanos a nivel de Bogota. *Acta Med Colomb.* 1982;7(6):461–466.

[18] Acevedo LE Solarte I : Gasimetria arterial en adultos jóvenes a nivel de Bogota. *Acta Med Colomb.* 1984;9(1):7–14.

[19] Sánchez MM : Valores de referencia de pH y gases arteriales en niños y adultos de San José, Costa Rica. *Revista Costarricense de Ciencias Médicas.* 1987;7(4):343–348.

[20] Vera CO : Valores normales de gases sanguíneos arteriales y del equilibrio acido base en la ciudad de La Paz – Bolivia. *Cuadernos.* 1991;37:18–27.

[21] Ortega H Millán A Mesa GE : Gasimetría arterial en población adulta sana de la ciudad de Medellín. *Acta Med Colomb.* 2002;27(2):98–102.

[22] Yumpo CD : Estudio de valores de referencia de gases arteriales en pobladores de altura. *Enfermedades del Tórax.* 2002;45:40–42.

[23] Hurtado JC Salazar T Peña M de la : Valores normales de ases arteriales en Bogotá. *Umbral Científico (Bogotá).* 2007;10:93–101.

[24] Maldonado D Gonzalez-Garcia M Barrero : Reference values for arterial blood gases at an altitude of 2640 meters. *Am J Respir Crit Care Med.* 2013;187:A4852.

[25] Pereira-Victorio CJ Huamanquispe-Quintana LE Castelo-Tamayo LE : Gasometría arterial en adultos clínicamente sanos a 3350 metros de altitud. *Rev Peru Med Exp Salud Publica.* 2014;31(3):473–479. 10.17843/rpmesp.2014.313.83 25418645

[26] Moina Veloz AP Villavicencio Barrezueta CP : *Valores de referencia de gasometría arterial en población adulta entre 18 Y 40 años de edad, residente a 2800 sobre el nivel del mar, en el Hospital Eugenio Espejo, en el periodo de Julio a Octubre del 2016 usando Normativa CLSI Ep28 – a3c, con metodología a priori.* Thesis, Universidad Central del Ecuador, Ecuador. 2016.

[27] Cárdenas-Santamaría F Ardila-Flórez M Jaramillo-Mejía M : Arterial blood gas in young adults at an average altitude of 1605-m above sea level: Armenia, Colombia 2016. *Rev Colomb Anaestesiol.* 2018;46:222–227. 10.1097/CJ9.0000000000000065

[28] Calderón Gerstein W López MO : Valores gasométricos en población adulta y adulta mayor residente de gran altitud. *An Fac Med.* 2020;81:154–160. 10.15381/anales.v81i2.18032

[29] Villacorta-Cordova F Carrillo Coba E Zubia- Olaskoaga F : Comparación de los valores normales de gases arteriales entre la altitud y el nivel del mar del Ecuador. *Revista de Medicina Intensiva y Cuidados Críticos.* 2020;13(2):88–91. 10.37463/intens-samay/010

[30] Santos-Martínez LE Arias-Jiménez A Quevado-Paredes J : Caracterización de parámetros del intercambio gaseoso en la Ciudad de México. *Rev Med Inst Mex Seguro Soc.* 2021;59(6):473–481. 34905321

[31] Zhuang J Droma T Sutton JR : Smaller alveolar-arterial O _2_ gradients in Tibetan than Han residents of Lhasa (3658 m). *Resp Physiol.* 1996;103:75–82. 10.1016/0034-5687(95)00041-0 8822225

[32] Li GH Zhang YQ Zhang HQ : Blood gas analysis of healthy people in Diqing Tibetan Autonomous Prefecture in Yunnan Province. *Ann Palliat Med.* 2021;10(1):285–291. 10.21037/apm-20-2206 33353353

[33] Loeppky JA Scotto P Charlton GC : Ventilation is greater in women than men, but the increase during acute altitude hypoxia is the same. *Respir Physiol.* 2001;125(3):225–237. 10.1016/S0034-5687(00)00221-8 11282389

[34] Paulev PE Zubieta-Calleja GR : Essentials in the diagnosis of acid-base disorders and their high altitude application. *J Physiol Pharmacol.* 2005;56(Suppl 4):155–170. 16204789

[35] Zubieta-Calleja G Zubieta-Castillo G Zubieta-Calleja L : Do over 200 million healthy altitude residents really suffer from chronic acid-base disorders? *Ind J Clin Biochem.* 2011;26(1):62–65. 10.1007/s12291-010-0088-9 22211016 PMC3068777

[36] Pagana KD Pagana TJ : *Mosby’s manual of diagnostic and laboratory tests.* 6th ed. St. Louis: Elsevier;2018.

[37] Merino MA Sahuquillo J Borrul A : ¿Es el lactato un buen indicador de hipoxia tisular? Resultados de un estudio piloto en 21 pacientes con un traumatismo craneoencefálico. *Neurocirugía (Astur).* 2010;21(4):289–300. 10.4321/s1130-14732010000400001 20725697

[38] Hochachka PW Beatty CL Burelle Y : The lactate paradox in human high-altitude physiological performance. *News Physiol Sci.* 2002;17:122–126. 10.1152/nips.01382.2001 12021383

[39] Villena JE : Diabetes mellitus in Peru. *Ann Glob Health.* 2015;81(6):765–775. 10.1016/j.aogh.2015.12.018 27108144

[40] Gonzales GF Tapia V : Hemoglobina, hematocrito y adaptación a la altura: su relación con los cambios hormonales y el periodo de residencia multigeneracional. *Rev Med.* 2007;15(1):80–93.

[41] Fernandez L : Dataset for “Arterial blood gas analysis of healthy residents in Huamachuco, Peru (3,164 m)”.[Dataset]. *figshare.* 2023. 10.6084/m9.figshare.22720630.v2 PMC10594050 37881331

[42] Fernandez L : STROBE checklist for “Arterial blood gas analysis of healthy residents in Huamachuco, Peru (3,164 m)”.[Dataset]. *figshare.* 2023. 10.6084/m9.figshare.22720723.v2 PMC10594050 37881331

