# Supplementary material for: Arterial blood analysis of healthy residents in Huamachuco, Peru (3,164 m): a cross-sectional study
Source: F1000Res. 2023 Sep 26;12:885. Originally published 2023 Jul 26. [Version 2] doi: 10.12688/f1000research.134567.2 (PMC10594050; doi:10.12688/f1000research.134567.2)
Supplement: Peer review history

# Reviewer report: Reviewer response for version 2

**DOI:** 10.5256/f1000research.156434.r209590

**Published:** 2023-10-23

**Authors:** Erica Heinrich

**Affiliations:** 1 University of California Riverside, Riverside, California, USA

I thank the authors for their careful attention to my comments. I have no further concerns.

Is the work clearly and accurately presented and does it cite the current literature?

Yes

If applicable, is the statistical analysis and its interpretation appropriate?

Yes

Are all the source data underlying the results available to ensure full reproducibility?

Yes

Is the study design appropriate and is the work technically sound?

Yes

Are the conclusions drawn adequately supported by the results?

Yes

Are sufficient details of methods and analysis provided to allow replication by others?

Yes

Reviewer Expertise:

High-altitude medicine, pulmonary physiology, control of breathing

I confirm that I have read this submission and believe that I have an appropriate level of expertise to confirm that it is of an acceptable scientific standard.

# Reviewer report: Reviewer response for version 1

**DOI:** 10.5256/f1000research.147631.r198146

**Published:** 2023-09-12

**Authors:** Niroj K. Sethy

**Affiliations:** 1 Defence Institute of Physiology and Allied Sciences (DIPAS), New Delhi, Delhi, India

The manuscript reports arterial blood gas analysis of 17 males and 29 females from Huamachuco, Peru (3,164 m). Though the rationale of the study is interesting, I have reservations for number of participants, combining both the genders, and conclusions.

1. Number of participants are on lower side. The authors must do a power analysis for such clinical studies ^1^.
2. The authors have excluded diabetic volunteers from the study. Hence, a comment like "lower prevalence of diabetes among highlanders...." is little misleading.
3. The conclusion statement "Northern Peruvians have and Andean-style adaptation to high altitude" based on arterial blood gas analysis a big claim.
4. In such field studies involving blood collection, I personally expect some other methods like ELISA/western blotting -based evidences for the original hypothesis.
5. It is now a well known fact that hypoxia response of males and females are distinct. Thus, combing male and female volunteer data sets for deciphering common hypoxia response is unwarranted.
6. Is there any effect of elevation (Study location section of result section) on blood gas parameters? What are the authors opinion about the same parameters at 2,200 m or 4,600 m (the lowest and highest elevations of the studied district)?

Is the work clearly and accurately presented and does it cite the current literature?

Yes

If applicable, is the statistical analysis and its interpretation appropriate?

Partly

Are all the source data underlying the results available to ensure full reproducibility?

Yes

Is the study design appropriate and is the work technically sound?

Partly

Are the conclusions drawn adequately supported by the results?

Partly

Are sufficient details of methods and analysis provided to allow replication by others?

Partly

Reviewer Expertise:

Hypoxia, High altitude biology, Human response to hypoxia

I confirm that I have read this submission and believe that I have an appropriate level of expertise to state that I do not consider it to be of an acceptable scientific standard, for reasons outlined above.

## References

[1] : Sample size estimation and power analysis for clinical research studies. *J Hum Reprod Sci* .2012;5(1) : 10.4103/0974-1208.97779 7-13 10.4103/0974-1208.97779 22870008PMC3409926

# Response

**Published:** 2023-09-22

**Authors:** Lissett Fernández

**Affiliations:** Physiology Laboratory, Universidad Privada Antenor Orrego, Trujillo, La Libertad, Peru

1. The authors agree that the study was small and that a larger sample size would have provided more reliable data. However, it is challenging to plan and execute a study in rural Peru, so this had to be considered. Power analyses are useful when two different groups are compared, but this paper describes a population sample. Nonetheless, we included a confidence interval calculation for the blood gas parameters in Table 1 and for diabetes prevalence. We think a confidence interval is a useful tool to describe how the data relates to the overall population’s blood gas parameters. Also, the cited article in this comment was retracted.
2. The authors excluded participants with diabetes from the arterial blood gas analysis section of the study but included them to discuss diabetes prevalence. Participants with diabetes were allowed to have their blood tested and were included in our discussion of diabetes prevalence.
3. Perhaps the authors were too bold in concluding Andean style adaptation. Instead, we have changed the wording to reflect uncertainty.
4. We recognize that additional experiments could have supported our claims, but the logistical difficulties involved with transporting an arterial blood gas monitor to a mountain town in Peru was stretching the capabilities of the research team.
5. Only data that did not show a significant difference using the Mann-Whitney U Test with Bonferroni correction between men and women were aggregated. Indeed, some parameters, like hematocrit and carbonate were different and were reported as different values for male and female participants (Table 1).
6. Most everyone in the study lived in the city of Huamachuco or were present there for a few days, so the resident altitude range isn’t as large as all the possible altitudes in Huamachuco. Furthermore, the tests were completed in the same location, controlling for elevation.

# Reviewer report: Reviewer response for version 1

**DOI:** 10.5256/f1000research.147631.r198140

**Published:** 2023-09-08

**Authors:** Erica Heinrich

**Affiliations:** 1 University of California Riverside, Riverside, California, USA

The goal of this study was to evaluate arterial blood gas values in healthy Northern Peruvians living at high altitude. Arterial blood was collected and analyzed in 46 participants. The main findings are that the arterial pH, O _2_, CO _2_, and other factors are comparable to the expected values at this altitude, but that Northern Peruvians had slightly elevated arterial PO _2_, potentially indicating adaptation to high altitude.

 While these findings do not substantially move the field forward, these are valuable measurements that contribute to our larger understanding of the effect of high altitude on arterial blood gas values. The data presented in Figure 1, from many different studies, can serve as a useful reference for others as well.

**Minor comments:**

 “However, these results were included in our analysis of the prevalence of diabetes and impaired fasting glucose.” How can this analysis be done if diabetes was an exclusion criteria? Clarify that these were exclusion criteria only for one part of all analyses.

 Can information be provided about how long participants had been at high altitude? Similarly, is the data in Figure 1 controlled for time spent at high altitude? These values will change with acclimatization and long-term adaptation.

 “The median blood pressure was 100/60 mmHg”. This is quite low for a median. Can the authors explain or speculate on why this may be?

 Figure 1: It is a bit difficult to see differences in the three point types for American, This Study, and Tibet. Can this figure be made in color or in some other way show the differences more clearly?

 I have some thoughts on the use of the term “Andean-style adaptation”. One could predict that some of the adaptations or maladaptations (such as excessive erythrocytosis and blunted ventilation/respiratory drive) in Andean high-altitude natives may be the normal response expected from other ancestral groups when exposed to a lifetime of high-altitude hypoxia, and not a specific phenotype expected only to appear in Andean individuals. However, since we don’t have much data from individuals of sea-level ancestry living for very long periods at high altitude, we can not test this idea yet. In my opinion, I would pull back from using this term and phrase this as something along the lines of “similar to adaptations seen in Andean high-altitude natives”, but it’s up to the authors.

Is the work clearly and accurately presented and does it cite the current literature?

Yes

If applicable, is the statistical analysis and its interpretation appropriate?

Yes

Are all the source data underlying the results available to ensure full reproducibility?

Yes

Is the study design appropriate and is the work technically sound?

Yes

Are the conclusions drawn adequately supported by the results?

Yes

Are sufficient details of methods and analysis provided to allow replication by others?

Yes

Reviewer Expertise:

High-altitude medicine, pulmonary physiology, control of breathing

I confirm that I have read this submission and believe that I have an appropriate level of expertise to confirm that it is of an acceptable scientific standard, however I have significant reservations, as outlined above.

# Response

**Published:** 2023-09-22

**Authors:** Lissett Fernández

**Affiliations:** Physiology Laboratory, Universidad Privada Antenor Orrego, Trujillo, La Libertad, Peru

We agree with the reviewer’s comments and recognize the limitations of this paper. It is our hope, too, that the compilation of blood gas results from the last several decades will be a useful reference for establishing reference parameters.

 Fasting arterial blood glucose was measured concurrently with blood gas. Therefore, the authors had access to blood glucose concentrations for all the participants for which a sample was drawn, even those who were later excluded from the blood gas results due to diabetes. Given that we still had the data, we felt reasonable to include it in an analysis of diabetes in the area to shed light on whether diabetes prevalence in Huamachuco was different than nearby lowlands. The authors recognize that this is somewhat difficult to follow, so the text was updated to be clearer about what participants were included at what stage of the study.

 One selection criterion was that the participant had to have resided in Huamachuco district for at least 5 years prior to the study, with no recent travel to lowland. As for the other papers cited, the authors tried, as much as possible, to include only residents, but each paper had their own criteria for what qualifies as resident. We added text in the conclusion that indicates that the results here are not applicable to sojourners to high altitude.

 The authors think that the blood pressure measured was reasonable, given the age of the study participants. A study from Cuzco, Peru, at a similar elevation, measured blood pressure to be 100/67 (Reference: Pereira, 2014). Additionally, a large study in Canada suggested a “normal” value for blood pressure in 20–29-year-old people to be approximately 105/68 ( https://www150.statcan.gc.ca/n1/pub/82-625-x/2016001/article/14657-eng.htm).

 From these data, and considering the characteristics of the group we studied, the authors think that the measured blood pressure is reasonable.

 We updated the figure to take advantage of color to make the different groups clearer.

 Perhaps we were too bold in concluding Andean style adaptation, so we changed the wording where appropriate to suggest a greater level of uncertainty.

# Reviewer report: Reviewer response for version 1

**DOI:** 10.5256/f1000research.147631.r198150

**Published:** 2023-09-04

**Authors:** Latika Mohan

**Affiliations:** 1 All India Institute of Medical Sciences, Rishikesh, utt, India

The article presents valuable data, is topical and useful. The study examines arterial blood gas analysis in adapted young adults residing at 3000m above sea level and discusses trends in ABGA with change in altitude. It is acknowledged that it is difficult to do such studies on normal subjects at altitude locations, but a larger sample size would have yielded more robust analysis. Was a sample size calculation done?

 The methodology has been described in detail and all procedures documented well. Although the authors admit that most of the ABGA parameters failed to reach statistical significance, the trends can be observed in table 1, and follow the expected trends for pO _2_, pCO _2_. In contrast the pH and Bicarbonate values do not seem to follow the inverse relationship. This needs to be discussed in greater detail, as these are long term residents and there may be physiological compensatory mechanisms coming into play.

 The regression model presented for predicting pO2 is very interesting, and larger population studies are required. As the altitude adaptation strategies are different in Tibetians and the Andeans, there may be merit in analysis of trends by ethnicity. 

 Overall, well written and useful study.

Is the work clearly and accurately presented and does it cite the current literature?

Yes

If applicable, is the statistical analysis and its interpretation appropriate?

Partly

Are all the source data underlying the results available to ensure full reproducibility?

Yes

Is the study design appropriate and is the work technically sound?

Yes

Are the conclusions drawn adequately supported by the results?

Partly

Are sufficient details of methods and analysis provided to allow replication by others?

Yes

Reviewer Expertise:

High altitude physiology, Neurophysiology

I confirm that I have read this submission and believe that I have an appropriate level of expertise to confirm that it is of an acceptable scientific standard.

# Response

**Published:** 2023-09-22

**Authors:** Lissett Fernández

**Affiliations:** Physiology Laboratory, Universidad Privada Antenor Orrego, Trujillo, La Libertad, Peru

A sample size calculation was not done before the experiment because, as the reviewer points out, it is difficult to recruit healthy participants. Furthermore, sample size and power calculations are used for studies that compare groups; this study simply reported values from one group. Instead of a sample size calculation, we include a 95% confidence interval for blood gas parameters measured.

 The authors did an additional analysis of the data and found there was no statistically significant correlation between actual bicarbonate concentration and arterial blood pH, but there was one between pCO _2_ and bicarbonate. These results and their discussion were added to the text.

 We attempted to separate Andean and Tibetan blood gas results in figure 1 (diamonds and circles). We could only find 2 reports conducted in Tibet, but we could certainly add more if we were made aware of them. We also updated the figure to include color to better contrast the different groups presented.
